# Decreased Frequency of Circulating Myelin Oligodendrocyte Glycoprotein B Lymphocytes in Patients with Relapsing-Remitting Multiple Sclerosis

**DOI:** 10.1155/2015/673503

**Published:** 2015-05-18

**Authors:** Annie Elong Ngono, Maud Lepetit, Markus Reindl, Alexandra Garcia, Flora Guillot, Athénaïs Genty, Mélanie Chesneau, Marion Salou, Laure Michel, Fabienne Lefrere, Kathrin Schanda, Berthe-Marie Imbert-Marcille, Nicolas Degauque, Arnaud Nicot, Sophie Brouard, David-Axel Laplaud, Jean-Paul Soulillou

**Affiliations:** ^1^INSERM, UMR1064, 44093 Nantes, France; ^2^Faculté de Médecine, Université de Nantes, 44035 Nantes, France; ^3^Service de Neurologie, CHU de Nantes, 44093 Nantes, France; ^4^Clinical Department of Neurology, Innsbruck Medical University, 6020 Innsbruck, Austria; ^5^INSERM, CIC 004, 44093 Nantes, France

## Abstract

Although there is no evidence for a role of anti-MOG antibodies in adult MS, no information on B lymphocytes with MOG-committed BCR is available. We report here on the frequency of anti-MOG B cells forming rosettes with polystyrene beads (BBR) covalently bound to the extracellular domain of rhMOG in 38 relapsing-remitting patients (RRMS) and 50 healthy individuals (HI). We show a substantial proportion of circulating anti-MOG-BBR in both RRMS and HI. Strikingly, MOG-specific B cells frequencies were lower in MS than in HI. Anti-MOG antibodies measured by a cell-based assay were not different between MS patients and controls, suggesting a specific alteration of anti-MOG B cells in MS. Although anti-MOG-BBR were higher in CNS fluid than in blood, no difference was observed between MS and controls. Lower frequency of MOG-BBR in MS was not explained by an increased apoptosis, but a trend for lower proliferative capacity was noted. Despite an efficient B cell transmigration across brain derived endothelial cells, total and anti-MOG B cells transmigration was similar between MS and HI. The striking alteration in MOG-specific B cells, independent of anti-MOG antibody titers, challenges our view on the role of MOG-specific B cells in MS.

## 1. Introduction

Multiple sclerosis is a chronic inflammatory disease characterized by leukocyte infiltration and white matter demyelination [1]. Among lymphocytes, T cells are prevalent in inflammatory lesions [2] and anti-myelin T cell frequency was found increased in MS patient blood [3]. Myelin antigen-specific T lymphocytes have attracted a great deal of attention due to their potential for induction by adoptive transfer experimental allergic encephalomyelitis (EAE), an animal model of the immune component of the disease [4–6]. However, myelin reactive T cells are also found in healthy individuals (HI) [3, 7]. Antibodies against myelin derived possible autoantigens, and particularly anti-MOG, have been widely studied with no clear evidence of linkage with the disease prevalence or severity in adult MS disease [8]. However, several recent studies indicated that antibodies to MOG are present in a subset of predominantly pediatric inflammatory demyelinating diseases different from MS such as ADEM or AQP4-IgG seronegative NMO (see [9] for review). A possible role of B cells in MS has been more recently highlighted through the beneficial clinical effect of anti-CD20 monoclonal antibodies which, before modifying antibodies titers, deplete B cells [10] and modify B cell functions [11]. Indeed, B cells present several functions thought to play a role in MS autoimmune processes [8]. Harp et al. have shown that, in MS, B cells committed to myelin proteins are efficient as antigen presenting cells [12]. B cells can also exhibit a regulatory function in autoimmune diseases [13, 14] or in a transplantation setting [15, 16]. Yet, an alteration of this function in MS [17] remains controversial [18]. B cell tolerance to autologous determinants depends on a first checkpoint which occurs in bone marrow [19]. Autoreactive B cells are nevertheless released in the periphery [20] where a T cell dependant second checkpoint operates. However, a substantial proportion of circulating B cells still remains poly- or cross-reactive despite these processes.

In this paper, using a novel approach [21] to detect MOG committed B cells in comparison with circulating anti-MOG antibodies, we show that (i) as for anti-MOG T cells [7, 22] normal individuals present a substantial high level of circulating anti-MOG B cells and (ii) although there were no differences in circulating anti-MOG antibodies, MS patients have a significantly lower circulating anti-MOG B cell frequency than healthy individuals.

## 2. Materials and Methods

### 2.1. Patients and Healthy Controls

Patients included in this study were diagnosed with MS using revised Mac Donald criteria [23]. 38 Relapsing-Remitting patients (RRMS), listed in Table 1, were recruited. RRMS group was composed of 27 females and 11 males ranging from 23 to 60 years old (mean age: 36.74). All patients were scored on the Kurtzke Expanded Disability Status Scale (EDSS) and were without immunomodulatory treatment for at least three months and immunosuppressive treatment for at least six months before testing. Another group of eight patients with secondary progressive MS, from 40 to 64 years old (mean age: 53.25) was also included with 6 females and 2 males. 50 healthy individuals (HI) participated in the study, 31 females and 19 males ranging from 22 to 61 years old (mean age: 38.24).

Nine patients with clinically isolated syndrome (CIS) and eight with other noninflammatory or inflammatory neurological disease (OND or OIND) were also enrolled in the intrathecal study. There were six females and three males ranging from 20 to 61 years old in the CIS group (mean age: 36.44). The control group (Table 1) was composed of four females and four males ranging from 15 to 77 years old (mean age: 40.13). Our study complies with the Nantes University Hospital Ethical Committee guidelines and all participants signed an informed consent for the study.

### 2.2. Obtention of Protein-Coupled Beads

Extracellular domain of recombinant human MOG_1-125_ (rMOG) (Eurogentec, France) expressed in* E. coli*, human albumin (LFB, France), and nontoxic tetanus toxin C-fragment (Sigma, France) proteins were coupled to fluorescent Bio-Plex COOH beads (Bio-Rad, France) as described [21]. The carboxyl groups of fluorescent COOH beads were activated by EDC (1-ethyl-3-[3-dimethylaminopropyl]carbodiimide hydrochloride) (Fisher Scientific, France) and S-NHS (sulfo-N-hydroxysulfosuccinimide) (Fisher Scientific); then the Bio-Plex amine coupling kit (Bio-Rad) was used to couple proteins to the activated COOH beads. The coupling reaction was systematically checked through flow cytometry using the appropriate antibodies. For MOG, we checked that the anti-MOG 8.18C5 mouse antibody, shown to react with the folded MOG pattern [24], effectively recognizes the MOG_1-125_ covalently coated beads. In addition, we checked that these MOG_1-125_ coated beads are also recognized by B cells from transgenic 8.18C5 antibody mice [21].

### 2.3. Frequencies and Phenotype of Blood MOG-Specific B Cells

Plasmas were collected from blood samples and mononuclear cells were isolated by Ficoll-Hypaque density gradient centrifugation (PAA, France) and frozen in serum-DMSO 10%. Purified B cells were obtained by negative magnetic selection (Miltenyi, France) from cryopreserved PBMC. Briefly, 3 × 10^5^ B cells were stained with CD19-PE (Clone HIB19) (BD, France), CD27-QDot 605 (CLB-27/1) (Invitrogen, France), IgD-FITC (IA6-2) (BD), CD5-FITC (53-7.3) (BD), CD38-PECy5 (Clone LS198-4-3) (Beckman Coulter, France), CD40 (5C3) (BD), KI-67-FITC (B56) and IgG1, к isotype control (BD) at 4°C for 30 minutes. The cells were then washed twice with PBS/2%FCS/2 mM EDTA buffer and incubated with protein-coated beads for 1 hour at 4°C and DAPI was added before FACS assay to select only the live cells. Specificity was tested through competitive incubation of the B cells with soluble MOG or albumin for 30 minutes prior to the addition of MOG-coated beads. The B cells were also preincubated with anti-IgG+IgA+IgM polyclonal Fab'2 fragments. Polyreactivity was also tested by the same approach. Briefly, soluble dsDNA (Sigma, France), LPS (Sigma), and insulin (Sigma) were added during 30 minutes on B cells prior to the incubation with MOG-coated beads. The frequency of antigen-specific B cells was evaluated on 3 · 10^4^ live CD19^+^ cells recorded for each sample through flow cytometry, using a LSR II (BD). All analyses were performed with FlowJo software, version 7.6.12 [21].

The absolute B-lymphocyte count was determined by combining a precise volume of blood with appropriate antibodies (CD45-V500—Clone HI30—and CD19-PE) in TruCount tubes (BD) for 15 minutes at room temperature. Then, the red blood cells were lysed with FACS lysing solution (BD). The TruCount tubes were analyzed through flow cytometry and the quantity of B cells/*μ*L of blood was obtained.

### 2.4. Apoptosis Assay

An annexin V-FITC apoptosis detection kit (BD) was used to evaluate the apoptosis in the BBR fraction. Briefly, 3 · 10^5^ purified B cells were stained with CD19-PE and incubated with protein-coupled beads for 1 hour. Annexin V-FITC was added and incubated with the cells for 15 minutes at room temperature (RT). Then, binding buffer and DAPI were added before analyzing by flow cytometry. Live B cells (annexin V^−^ DAPI^−^), early apoptotic B cells (annexin V^+^ DAPI^−^), and late apoptotic B cells (annexin V^+^ DAPI^+^) were selected.

### 2.5. Quantification of Intrathecal MOG-Specific B Cells

After lumbar puncture, 10 mL of CSF was immediately added to a RPMI supplemented medium. The CSF samples were centrifuged at 1500 rpm for 7 minutes to collect the cells and supernatants were collected. Cell pellets were resuspended in PBS/2%FCS/2 mM EDTA buffer and stained with CD19-PE antibody for 30 minutes at 4°C. The CSF cells were then washed and incubated with either albumin or MOG-coupled beads as indicated above.

Out of 29 CSF samples obtained during the study period, only 17 CSF (58%) patients contained more than 50 live CD-19 reactive B cells per sample, the minimal amount considered required for data interpretation. The total cell numbers for these CSF ranged from 50 to 2710 live B cells. Blood samples were obtained from each CIS and control patient, for comparison (9 from CIS and 8 from non-MS).

### 2.6. B Cell Transmigration Assay through Endothelial Cell Line

The human blood-brain-barrier (BBB) endothelial cell line HCMEC/D3 is an immortalized endothelial cell line derived from a primary cell culture coexpressing hTERT and the SV40 large T antigen via a lentiviral vector system [25]. Transmigration assays were performed using the Transwell system (8.0 *μ*m pore filters; BD Falcon, France) as previously described [26]. Cell concentration was tested and concentrations which allowed more than 5% human albumin diffusion in the lower chamber after 6 hours were discarded. Two days before the migration assay, 1 × 10^6^ HCMEC/D3 cells were cultured on the apical side of the filter insert. All cells were grown in endothelial basal medium-2 (EBM-2, Lonza) supplemented with human serum (PAA). 5 × 10^5^ B cells were added on top of the BBB layer. After 18 h at 37°C with 5% CO_2_, the contents of the bottom chamber were collected with EDTA to detach any adherent cell, and B cells were counted. The transmigrated cells were washed, stained with CD19-PE, and incubated with albumin or MOG coupled beads as indicated previously.

### 2.7. Assessment of Anti-MOG and EBNA1 Reactive Circulating Antibodies

Anti-MOG antibodies were assessed with an immunofluorescent* cell-based assay* (CBA) as described in detail elsewhere [27]. Briefly, all plasma and CSF samples were tested for reactivity against the human MOG expressed in HEK293 cells using an immunofluorescence live cell assay. The plasma samples were tested at a 1/20 dilution and CSF samples were undiluted. Titers were considered positive above a cut-off of 1 : 160 [27]; correlations were also performed using direct OD values.

The detection of anti-EBNA1 IgG in plasma samples was performed routinely using the DiaSorin kit (Liaison EBNA1 IgG) on an automat (Liaison XL).

### 2.8. Statistical Analysis

All data are expressed as mean ± SEM. Mann-Whitney tests were used to compare pairs of different groups and Wilcoxon test to compare two variables in the same group. *t*-test was used to compare pairs of groups with more than 30 individuals. One way ANOVA or Kruskall Wallis tests were used to compare more than 2 groups. All statistical analyses were performed with GraphPad Prism 5. Results were considered significant when *P* < 0.05.

## 3. Results

### 3.1. Validation of the Method and Reactivity of the 8.18C5 Antibody

Mouse monoclonal antibody 8.18C5 recognizes conformational MOG_1-125_ (Ig-like domain) but not the linear MOG epitopes in mice [24]. 8.18C5 antibody was used to both check the efficiency of the MOG coupling to the beads and the covalently bound MOG reactivity to the antibody. The reactivity of the MOG coated beads with the 8.18C5 antibody was potent (91%) as shown in Supplementary Figure 1a available online at http://dx.doi.org/10.1155/2015/673503. The MOG covalently coated beads were also recognized by the transgenic IgH-MOG B cells (corresponding to the 8.18C5 heavy chain) [28]. Spleen B cells of these mice exhibited 59% of MOG-BBR, with a drop of 90% when the B cells were preincubated with soluble rMOG but not with albumin (Supplementary Figure 1b).

### 3.2. Quantification of MOG-Specific B Cell Frequencies in MS Patients

**Figure 1:**
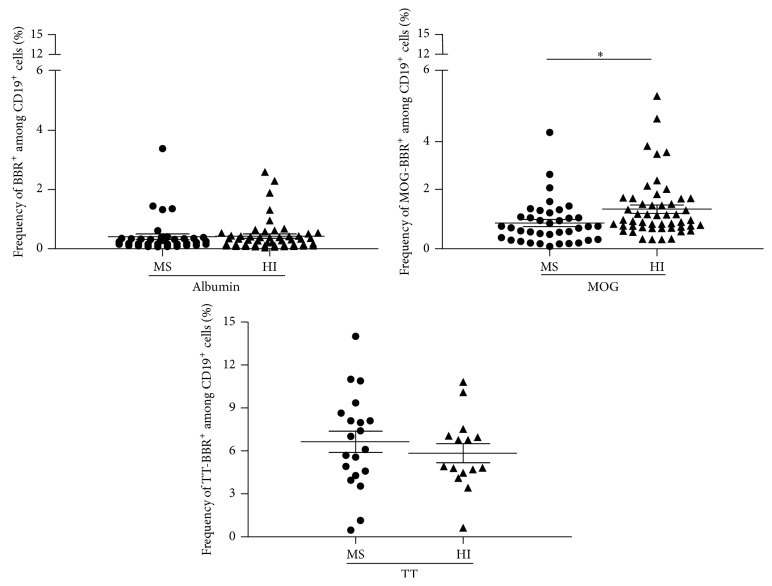
Quantification of MOG-specific B cell frequencies in MS patients and HI. Purified B cells, stained with CD19 antibody, were incubated with human albumin, rMOG, or tetanus toxin (TT) coated beads. After exclusion of dead cells (DAPI^+^), the frequency of B cells which recognized albumin or rMOG was obtained in MS patients (*n* = 38) and HI (*n* = 50) ^*^ *P* = 0.0188,* unpaired t-test*. The frequency of B cells bound to TT coated beads was also assessed in MS patients (*n* = 20) and in HI (*n* = 15) (ns, *P* > 0.05,* Mann-Whitney test*).

**Figure 2:**
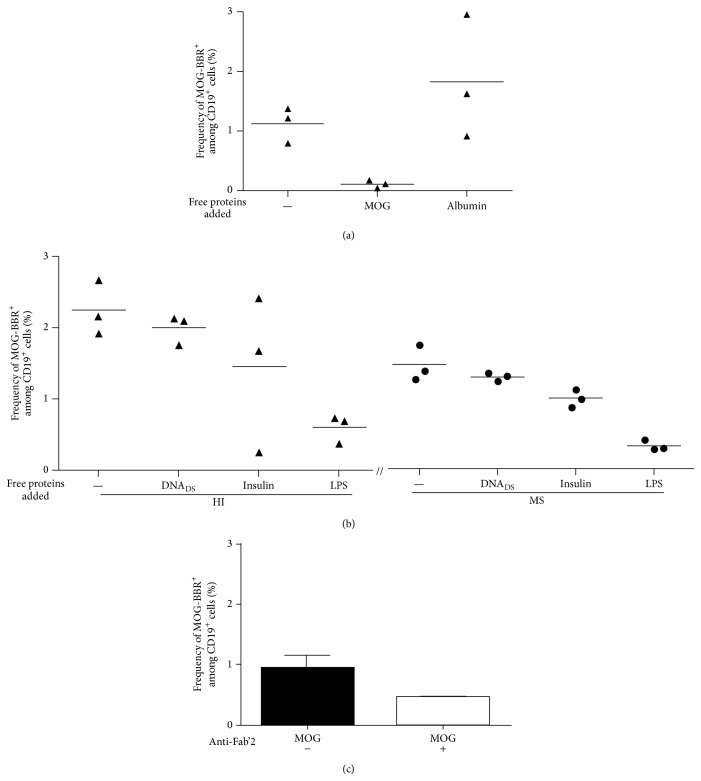
Reactivity of MOG-specific B cells. B cells were preincubated with 20 *μ*g of soluble MOG, albumin, dsDNA, insulin, or LPS before the addition of MOG-coated beads (*n* = 3). (a) The frequency of MOG-BBR drops with the soluble MOG preincubation but not with albumin preincubation. (b) The recognition of MOG-coated beads decreased after the addition of soluble LPS (% of inhibition: 73%), dsDNA (11%), and insulin (35%). Competitive assay was tested in MS (*n* = 3); the percentage of inhibition after soluble antigen addition was the same proportion as HI: LPS (78%), dsDNA (11%), and insulin (32%). (c) B cells were preincubated with Fab'2 anti-human IgG+IgA+IgM. The frequency of B cells recognizing MOG-coated beads was assessed on 3 HI. Fab'2 antibody modified the frequency of MOG-specific B cells (ns, *P* > 0.05,* Wilcoxon test*).

MOG is a myelin antigen only expressed in the CNS, which induces EAE, and is suspected to play a role in MS [29]. In this study, MOG-coated polystyrene beads were used to identify* in vitro* CD19^+^ cells able to make rosettes with the MOG coated beads (*referred to as BBR for bead/B cell rosettes*) as described elsewhere [21]. Several negative controls were used: uncoated beads, albumin coated beads, or T cells instead of B cells. Tetanus toxin (TT) coated beads were used as the positive controls [30]. Using this approach, we observed substantially high frequencies of circulating B cells rosetting with rMOG coated beads in both cohorts (MS patients and HI) (Figure 1). However, unexpectedly, the frequency of anti-MOG B cells was statistically lower in RRMS (*n* = 38) than in the cohort of 50 healthy individuals (HI) tested in parallel (0.86 ± 0.12 and 1.33 ± 0.14%, resp., *P* = 0.0188, Figure 1). In contrast, the frequency of B cells which recognized albumin coated beads was not statistically different in MS (0.40 ± 0.09%) and HI (0.42 ± 0.07%) (Figure 1). RRMS patients and HI show a similar and high frequency of B cells recognizing TT coated beads, 6.64 ± 0.74 and 5.84 ± 0.66%, *P* = 0.37 (Figure 1). The specificity and the polyreactivity of the MOG-coated beads were assessed by addition of 20 *μ*g of soluble rMOG, albumin, dsDNA, LPS, and insulin. The addition of soluble rMOG resulted in a dose dependent inhibition of MOG-specific B cell frequency (from 1.12 ± 0.17 to 0.11 ± 0.03) whereas soluble albumin did not provide competition (Figure 2(a)). Soluble LPS dropped by 73% the recognition of MOG-specific B cells (from 2.24 ± 0.22 to 0.59 ± 0.11). However, dsDNA (from 2.24 ± 0.22 to 1.99 ± 0.12) and insulin (from 2.24 ± 0.22 to 1.45 ± 0.63) have a smaller competing effect on the MOG-specific B cell frequency (Figure 2(b)). Polyreactivity of MOG-specific B cell in MS patients and HI was similar. MOG-specific frequencies after dsDNA and insulin incubation were, respectively, 1.30 ± 0.03 (11% of inhibition) and 0.99 ± 0.07 (32%) in MS group (Figure 2(b)).

The Fab'2 fragment anti-human Ig (A, G, and M isotypes) was also able to decrease the MOG-specific B cell frequency (nearly 50%) (Figure 2(c)), further suggesting that the interaction of the tested B cells with MOG-coated beads involved the surface membrane BCR.

**Figure 3:**
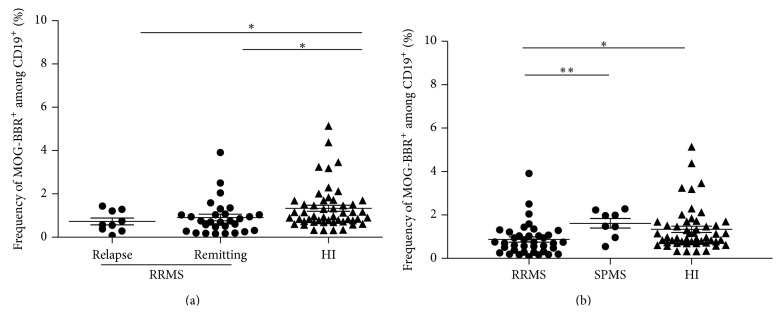
Quantification of MOG-specific B cell frequencies in MS patients according to disease activity and MS forms. (a) RR patients were classified according to disease state, for example, relapse or remission. We analysed the difference in MOG-BBR frequencies for patients in relapse (*n* = 9) and patients in clinical remission (*n* = 29) versus HI (*n* = 50) (1*P* < 0.05),* Kruskal-Wallis test*. No difference between patients in relapse and patients with clinical remission was observed (ns, *P* > 0.05). (b) Eight patients with secondary progressive (SP) forms were included and compared to RR patients and HI. There was a statistically significant difference between SPMS patients and RR MS patients (*P* < 0.05) but not between SPMS patients and HI (*P* > 0.05),* Kruskal-Wallis test*.

The frequency of MOG-BBR cells in MS was then classified according to the state of the disease and to the clinical form. No significant difference between the frequency of MOG-BBR of patients in relapse (*n* = 9, 0.72 ± 0.15%) and in clinical remission (*n* = 29, 0.92 ± 0.16%) was observed (Figure 3). Both groups had a lower frequency of MOG-BBR compared to HI (*P* = 0.01 and *P* = 0.02, resp.). In the same condition to detect MOG-specific B cells, a small group (*n* = 8) of patients with a secondary progressive form of MS (SPMS) showed a similar MOG-BBR frequency to the HI (1.61 ± 0.21 and 1.33 ± 0.14%, resp.,* ns*). The difference between the values of MOG-BBR for the SPMS and RRMS groups was statistically significant (*P* = 0.005).

### 3.3. Phenotype of MOG-BBR Cells

#### 3.3.1. Naïve, Memory Phenotype and Activation State

CD27 is a marker of human memory B cells [31]. The absolute value of B cells was assessed in RRMS patients and HI and no significant difference was observed (data not shown). Moreover, we find the same proportion of memory B cells in both groups (Supplementary Figure 2a). Assessment of frequency of memory (CD27^+^) and naïve (CD27^−^) B cells recognizing MOG-coated beads indicates 32.6 ± 3.16% CD27^+^ MOG-BBR and 64.86 ± 2.88% of CD27^−^ MOG-BBR in MS patients versus 37.31 ± 3.84% of CD27^+^ MOG-BBR and 59.77 ± 4.02% of CD27^−^ MOG-BBR in HI (Supplementary Figure 2b). The frequency of switched and unswitched memory MOG-BBR, as approached by CD27^+^IgD^−^ and CD27^+^IgD^+^ distribution, did not differ between MS and HI (Supplementary Figure 2c). Naïve MOG-BBR were more frequent than the memory phenotype in both groups (*P* = 0.01). In this compartment, activated naïve B cells (CD27^−^CD38^+^) were more represented than mature naïve B cells (CD27^−^CD38^−^) in MOG-BBR subsets (Supplementary Figure 2d).

The B cells were also stained for CD40 activation marker, a candidate for contributing to autoimmune processes in which B cell activation may play a role [32]. We assessed the CD40 MFI of MOG-specific B cells in MS and HI. No significant difference was observed between RRMS (*n* = 12) and HI (*n* = 11) in the MOG-BBR subset (Supplementary Figure 2e).

#### 3.3.2. Proliferation State

**Figure 4:**
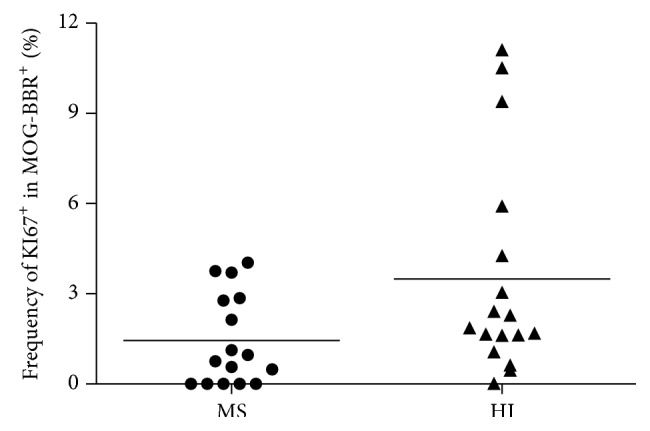
KI-67 expression in MOG-specific B cells: KI-67 marker was used to determine the proliferating states of MOG-BBR. The frequency of MOG-BBR positive for KI67 in MS (*n* = 16) was not different to HI (*n* = 17), (ns, *P* = 0.06)* Mann-Whitney test*.

Ki-67, expressed during all active phases of the cell cycle [33], was used to assess the proliferative status of MOG-specific B cells in RRMS patients and HI. Although the detection of KI-67 was difficult in unstimulated B cells, a trend (*P* = 0.06) was found for the two cohorts: 1.44 ± 0.37% MOG-BBR positive for KI-67 in MS patients (*n* = 16) and 3.49 ± 0.86% in HI (*n* = 17) (Figure 4). MOG-specific B cells were stained more strongly by KI-67 than B cells in MS and HI (*P* < 0.05, data not shown).

### 3.4. Apoptosis of Anti-MOG-BBR Cells

**Figure 5:**
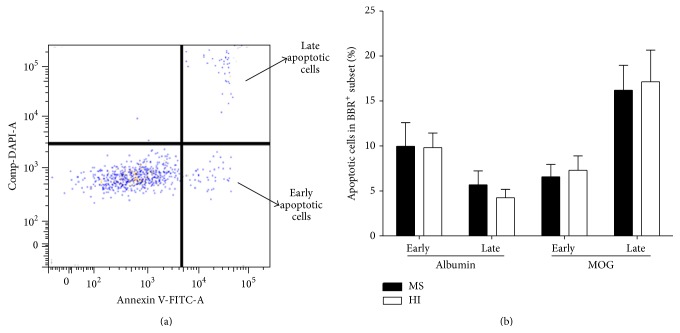
Analysis of apoptosis in MOG-specific B cells. (a) We used annexin V and DAPI to characterize MOG-BBR apoptosis cells in MS (*n* = 10) and HI (*n* = 10). We analysed annexin V^+^ DAPI^−^ (early apoptotic cells) and annexin V^+^ DAPI^+^ (late apoptotic cells) in the MOG-BBR subset. The gating strategy was represented. (b) We compared the frequency of MOG-BBR apoptotic cells in MS and HI in early (ns, *P* > 0.05) and late states (ns, *P* > 0.05),* Mann-Whitney test*.

Among several possible explanations for the decreased frequency of MOG-BBR in MS patients compared to HI, apoptosis was considered. A combination of annexin V (recognizing phosphatidylserine (PS) on the cell surface) and DAPI was used to detect early (annexin V^+^, DAPI^−^) and late (annexin V^+^, DAPI^+^) apoptotic cells (Figure 5(a)). Ten patients with RR-MS and 10 HI were tested. Early and late apoptosis markers of MOG-BBR showed no difference in MS and HI (for early apoptosis: 6.56 ± 1.38 and 7.29 ± 1.61%, *P* = 0.88; for late apoptosis: 16.20 ± 2.75 and 17.14 ± 3.53%, resp., Figure 5(b)).

### 3.5. Intrathecal MOG-Specific B Cells

**Figure 6:**
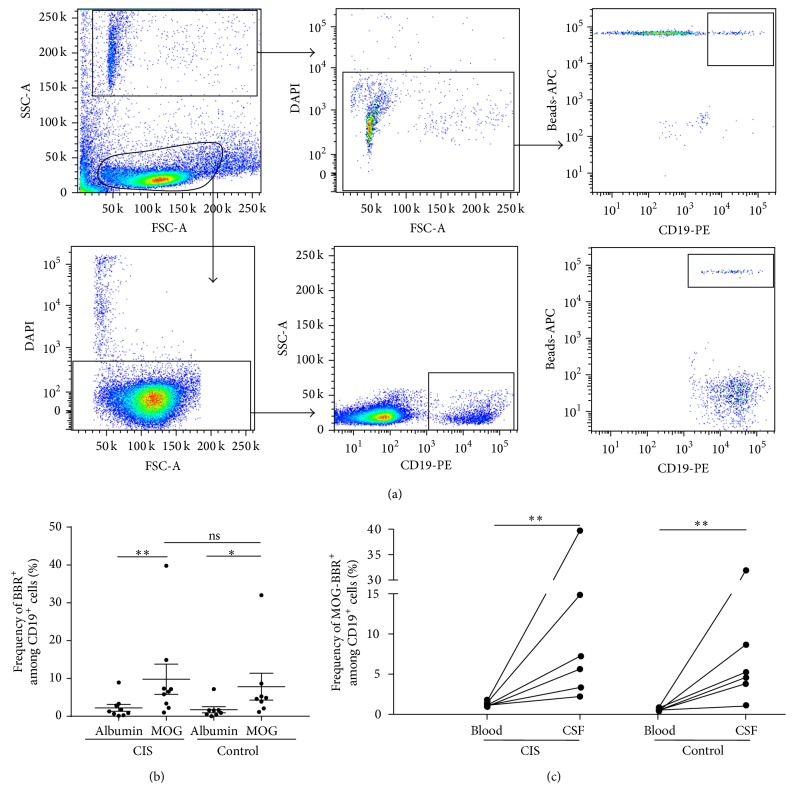
Intrathecal MOG-specific B cells in CIS and patients with non-MS-related pathologies. (a) The gating strategy of CSF analysis is represented. The number of B cells in this CIS sample was 1771 cells. The BBR frequency was obtained by FlowJo analysis which calculated the percentage of MOG-specific B cells among B cell subsets: 107 BBR corresponds to 5.69%. (b) Cells from fresh CSF samples were stained with CD19 antibody and incubated with rMOG and human albumin coated beads. After exclusion of dead cells (DAPI^+^), the frequency of BBR recognizing rMOG and albumin was assessed in CIS (*n* = 9) and control patients (*n* = 8) (ns, *P* > 0.05).* Mann-Whitney test and Wilcoxon test* were used to compare albumin-BBR and MOG-BBR in CIS (*P* < 0.05) and control patients (*P* < 0.05) (c). The frequency of circulating and intrathecal MOG-BBR was assessed in CIS (*n* = 6) and control patients (*n* = 6). Intrathecal frequency of MOG-BBR was higher than circulating frequency (ns, *P* > 0.005,* Mann-Whitney test*).

Although cell numbers in CSF samples reduced intrathecal study, we then checked if MOG-specific B cells accumulated in the spinal fluid of 8 patients with a clinically isolated syndrome (CIS), in whom there was a clinical need for CSF analysis and in 8 patients with other neurological disorders, both inflammatory and noninflammatory, as controls. All analyses were performed with CSF immediately processed after harvesting. As mentioned in the M&M, only 58% of the samples contained enough cells to perform the test. There was an average of 525 ± 105 B cells (CD19^+^DAPI^−^) in fresh CSF samples (ranging from 50 to 2710 B cells) in the tested samples. An example of the gating strategy is represented in Figure 6(a). Cells in CSF samples were selected according to the SSC and FSC parameters as beads and rosettes which had a small size (FSC) and high SSC. DAPI and CD19 markers were first used to analyze only live B cells and exclude dead rosettes. The costaining of APC and PE determines MOG-specific B cells (beads-APC/CD19-PE). Then, the Boolean gate function was used to determine the % of rosettes selected in CD19 live cells, giving the frequency of MOG-specific B cells.

We found an identical intrathecal frequency of MOG-specific B cells in CIS (10.28 ± 4.48%) and control individuals (8.62 ± 3.98%) (*P* = 0.6) (Figure 6(b)). The CSF frequency of MOG-BBR was higher in both groups compared to the albumin-BBR frequency in CIS (Figure 6(b)) and circulating MOG-BBR (1.28 ± 0.11 and 0.57 ± 0.10%) in CIS patients and in the control group, respectively (*P* < 0.05) (Figure 6(c)).

### 3.6. Anti-MOG-BBR Transfer through Brain-Derived Endothelial Cells

**Figure 7:**
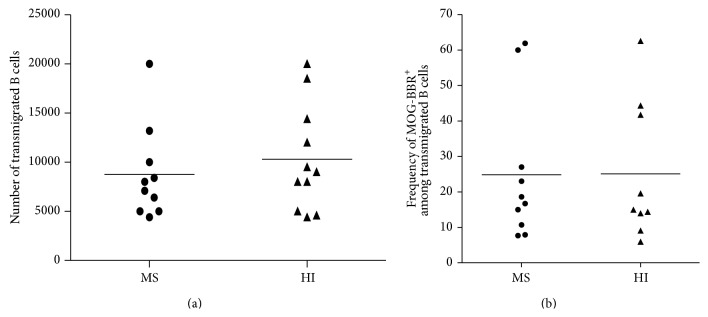
Transmigration of B cells across brain vessel derived endothelial cells. (a) B cells were negatively selected and a transmigration assay across HCMEC/D3 cell line was performed. Transmigrated cells in MS (*n* = 10) and HI (*n* = 11) were counted; no difference was observed (ns, *P* > 0.05,* Mann-Whitney test*). (b) Transmigrated B cells were stained and incubated with MOG coated beads. The frequency of MOG-BBR was assessed in MS patients (*n* = 10) and HI (*n* = 9) (ns, *P* > 0.05,* Mann-Whitney test*).

We first tested whether B cells from RRMS patients transmigrated more efficiently than B cells from HI across the endothelial cell layer* in vitro*. In MS, 8750 ± 1505 B cells transmigrated overnight compared to 10309 ± 1622 B cells in HI out of 5 × 10^5^ CD19^+^ cells initially placed in the double chamber device (about 2% of transmigrating B cells). Despite confirming that human B cells migrate efficiently across the blood-brain barrier [34], there was no statistical difference between the MS and HI values (*P* = 0.49, Figure 7(a)). We then checked whether MOG-BBR accumulated more efficiently in MS patients compared to HI by assessing the capacity of the B cells to make MOG-BBR after a transmigration assay. We observed no statistical difference in the transmigration rate of MOG-specific B cells in MS (24.85 ± 6.32%) and in HI (25.11 ± 6.50%) (Figure 7(b)).

### 3.7. Plasma Anti-MOG IgG Reactivity

The presence of anti-MOG antibodies was tested with a cell-based assay (CBA). A cut-off of 1 : 160 was chosen and used for discrimination of ADEM and other groups, as in previous studies [27]. Above this threshold, we found only two seropositive samples in a single RR MS patient (Supplementary Figure 3a). Surprisingly, one normal individual also exhibited a strong reaction. The frequency of BBR-MOG for this patient was 0.32%, which is not within the highest frequency values of RR MS patients. Similar observation can be done for the anti-MOG seropositive HI. We did not observe a significant correlation between MOG-specific BBR and CBA reactive soluble antibodies (*r* ^2^ = 0.032, *P* > 0.05) (Supplementary Figure 3b). Since beads were coupled to the extracellular domain of MOG, reactivity to this antigen was also analyzed by ELISA, but again no differences were observed between MS patients and controls and no significant correlation between MOG-specific BBR and MOG reactive soluble antibodies was found (data not shown).

The presence of anti-EBNA1 IgG, reported to be associated with MS [35, 36], was tested to see whether the frequency of MOG-specific B cells correlates with EBNA-1 titers. Anti-EBNA1 IgG titers were higher in MS patients than HI (*P* = 0.03) (Supplementary Figure 4) but no correlation was evidenced between the frequency of MOG-specific B cells and anti-EBNA1 titers (data not shown).

## 4. Discussion

Despite the beneficial effect of anti-CD20 in treating the disease, the potential role of B cells in multiple sclerosis (MS) has been less well documented than that of T cells. In this paper, we explore the frequency of MOG-specific B cells in the blood of patients with MS and in the spinal fluid of CIS patients, using a novel method based on the measurement of a direct interaction between a B lymphocyte and a fluorescent polystyrene bead to which human MOG is covalently bound [21].

The extracellular Ig-like MOG domain is unique in that it is the only protein structure known to induce both demyelinating autoantibody and encephalitogenic T cell responses in EAE [37]. MOG is a quantitatively minor component of myelin in CNS [38]. Antibodies directed toward the conformational MOG_1-125_ and not directed toward the linear MOG, such as the mouse antibody 8.18C5 [24], are instrumental in inducing anti-MOG antibody mediated EAE [39]. Several studies suggest that they are present in a subset of predominantly pediatric inflammatory demyelinating diseases which are distinct from MS [9]. While a significantly higher level of anti-EBNA1 titers was found in MS patients, no correlation with anti-MOG-BBR frequency was observed. Regarding the rMOG used, it is important to note that the 8.18C5 antibody recognizes 91% of the MOG_1-125_ after covalent binding to the beads, suggesting that a good proportion of the bound MOG_1-125_ exhibit the correct folding. However, to what extent the folding of MOG_1-125_ affects the frequency of B cells with a membrane antigen receptor interacting with MOG-coated beads could not be precisely assessed in this test, which differs from the cell-based assay used to measure soluble anti-MOG antibodies. Finally, since we did not test antibody secretion of MOG-BBR, we cannot exclude that this subset is able to produce some MOG antibodies. Nevertheless, it is important to note that we observed a significant difference in anti-MOG_1-125_ committed B cells between the MS patients and the controls (*P* = 0.018), whereas no difference in anti-MOG antibody titers against conformational and linear MOG has been found [40] in adult MS patients, clearly suggesting a B cell specific alteration in this disease.

A first finding was that the frequency of B cells engaging with MOG-coated beads was unexpectedly high, both in the MS patients and in healthy individuals (HI). This observation has been in parallel with the well documented description of high frequency of MOG-specific T cells in HI [7]. It is likely that B cell specific maturation processes are involved in the high anti-MOG B cell frequencies.

BCR cross-reactivity and/or polyreactivity studies of blood B cells [19] have indeed ambiguously shown a substantial percentage of circulating B cells (20% of immature B cells remain autoreactive, with 4% of them being polyreactive cells) having escaped the first BCR-dependant bone marrow selection checkpoint, which triggers apoptosis or reediting for autoreactive BCR [19, 20]. However in these seminal studies, the end point was the production of Ig against a panel of antigens in limiting conditions. A study of the direct binding of the B cell to its antigen may be a more sensitive tool for frequency calculation. We also tested the polyreactivity of MOG-BBR to dsDNA, LPS, and insulin antigens [19]. However, unexpectedly high values of competition for anti-MOG BCR were found for LPS, a major component of the outer membrane of Gram negative bacteria, and a toll-like receptor agonist. Cross-reactivity of surface BCR of MOG-BBR for dsDNA was roughly of the same magnitude as the cross-reactivity/polyreactivity of secreted antibodies anti-dsDNA after the first repertoire checkpoint of B cells [19]. Higher competition was noted for insulin. These differences may be due to a difference in maturation of surface B cell receptor that we explored versus secreted antibodies [19]. This polyreactivity normally downregulated in the last phase of B cell development has been proposed as the link between infection and autoimmune diseases leading to a breakdown of the immunological tolerance as documented in clinical settings and in animal models [41, 42]. In line with this, a study has shown an increase of immune reactivity to bacterial LPS in NMO patients [43]. However, our study was not designed to specifically address this issue and this observation requires confirmation and caution in interpretation.

In addition, our method used Luminex beads which, when used for testing specific antibodies such as anti-HLA, exhibits a gain of sensitivity of an order of magnitude compared to classical methods [44]. The case that an assay using the same readout (FACS) and material (Luminex) also displays a gain in sensitivity is conceivable.

Strikingly, we found a significant decreased MOG-specific B cell frequency in the blood of MS compared to age/gender matched control individuals. Importantly, the samples from HI and MS were routinely processed back to back in the tests. Furthermore, no difference was exhibited in the frequencies of B cells recognizing tetanus toxin and albumin coated beads between patients with MS and HI. Finally, the anti-MOG B cell frequency was decreased neither in a small group of MS patients with secondary progressive form (SPMS) nor in the CIS patients suggesting that this alteration occurs in the course of the relapse-remitting disease rather than at its onset. We did not find evidence for a specific phenotype bias of the anti-MOG B cells, with normal distribution (both for MS and HI) of naïve, memory cell phenotypes.

Recently, a subset of potentially autoreactive B cells, B1 cells, defined as CD3^−^CD20^+^CD27^+^CD43^+^ [45], was found to be lower in the blood of MS patients versus HI [46]. Although no test for the MOG specificity of the B1 cells was carried out in this study, the magnitude of the decrease in B1 cells (29% of the B cells) in the referred study was however too small (1.2% in MS patients versus 1.7% in normal individuals) to account significantly for our observation (RRMS had a 36% decrease in MOG-BBR). Furthermore, the B cell selection kit that we used did not select CD43^+^ cells, which accounted for most of the B1 cells. To the best of our knowledge, only one study is available on anti-MOG B cell frequency, using biotinylated MOG in a small cohort (*n* = 9) of patients with RRMS [12]. This interesting study suggests that circulating memory anti-MOG B cells of MS patients could provide a greater help to CD4^+^ T cells than memory anti-MOG B cells of HI. However, no significant difference of frequency in anti-MOG B cells between MS patients and HI was noted in this study. The reason for this discrepancy with our study is not clear, although our cohort was 4 times bigger (38 MS and 50 HI). Recently, another study reported that autoreactive B cells in MS were more prone to produce polyreactive antibodies to an extract of white matter [47] than in HI. However, no frequency of myelin or MOG-specific B cells was analyzed. Finally, our data do not support that idea that normal individual B cells have no reactivity against brain extracts, as was recently suggested [48]. However, it must be said that Kuerten et al. used* in vitro* polyclonally stimulated B cells and CNS extract.

Reasons for the significant decrease in anti-MOG circulating B cells remain so far undefined. Particularly, we could not find evidence for an increase in early or late apoptosis of anti-MOG B cells. However, the growth potential of MOG-specific B cells, as assessed by expression of the KI-67 marker, shows a trend (*P* = 0.06) for a lower expression in MS patients that may contribute to the lower frequency of anti-MOG-BBR in MS. The possibility that the “missing” MOG-specific circulating B cell population could be in the CNS was suggested by a documented increase of anti-MOG IgG in spinal fluid from MS patients [49] and by the shared BCR clonotypes between blood and CSF [50]. We could only measure the level of anti-MOG B cells in the CSF of CIS patients. However, we could not document a possible increased homing of MOG-specific B cells in CSF of our RR-MS patients since there is no clinical indication for CSF analysis in overt MS disease, making the study unethical.

Connections between the periphery and CNS allowing trafficking of antigens and presenting or effector cells have been well documented [51, 52] and may offer a substratum for transfer and homing of anti-MOG B cells in the brain. More recently evidenced connecting pathway may also be involved [53, 54]. Nevertheless, there was no difference of anti-MOG-BBR in transmigration assays although B cells (2%) showed efficient transmigration through a brain-derived endothelial cell layer, in concordance with another study [34].

In conclusion, our study gives the initial evidence of an abnormal circulating pool of anti-MOG B cells.* In vitro* analyses were unable to detect discrete changes in surface molecules involved in B cell homing in noncirculating sites of the immune system. It is possible that the mechanisms that govern this decreased MOG-specific frequency are no longer active at the time of the assay. Significantly, our observation involved a specific B cell alteration which is independent of the pattern of anti-MOG antibodies and which is not restricted to the optimal MOG conformation as is the case for soluble antibodies detected in the cell-based assay [9]. Recently, a cellular involvement of B cells, independent of their humoral function, was shown in pathogenesis of EAE [55]. Finally, whether MOG-specific B cells are actually involved in the disease pathology is not directly confirmed by our observations, considering that a high percentage of these specific B cells are also observed in HI blood, mimicking the situation described for T cells with myelin autoreactivity [3, 7]. In this respect, the positive effect of anti-CD20 on the disease outcome might be misleading in terms of the actual role of MOG-specific B cells in the disease. Indeed, besides the destruction of a fraction of potentially pathogenic B cells (partial depletion in lymph nodes [11]), such reagents also affect the properties of bystander B cells and may induce these cells to produce more immunoregulatory cytokines or to exhibit modified functions, as recently suggested [11, 56].

## 5. Conclusion

In this paper, we detected MOG-specific B cells in MS by a novel approach using fluorescent beads covalently bound to extracellular domain of MOG. We show for the first time that, as for T cells, healthy individual blood harbors autoreactive MOG B cells. In addition, we showed a significantly lower frequency of MOG-specific B cells in patients with relapsing-remitting MS compared to HI. The decrease of this subset suggests their implication in MS and stresses further studies.

## Supplementary Material

In this supplementary material, complementary figures are added to provide further information and to highlight results obtained in the study. Supplementary material corresponds to 1) Reactivity of the 8-18 C5 antibody for MOG; 2) Phenotype of B cells and MOG BBR subsets in MS and HI; 3) Anti-MOG antibody detection and; 4) Anti-EBNA1 antibody detection. Each enumerated figure content is detailed in legend and refered in the main text as supplementary figure.

## Figures and Tables

**Table 1:** Summary of patients and sample characteristics.

	*n*	Age	% females	% relapses	EDSS
RR MS	38	36.74 ± 9.8	71%	24%	1.75 ± 0.33
SP MS	8	53.25 ± 7.86	75%	—	—
HI	50	38.18 ± 11.48	62%	—	—
CIS	9	36.44 ± 14.82	67%	—	—
Controls	8	40.13 ± 21.70	50%	—	—

38 patients with relapsing-remitting form, 50 healthy individuals, and eight patients with secondary progressive form were included in this study. Forintrathecal analysis, nine patients with clinically isolated syndrome and eight controls were included. Among the eight controls without neurological disease, there was four hydrocephalus, one anti-N-methyl-D-aspartate (NMDA) receptors' encephalitis, one leukopathy, one tetrapyramidal syndrome, and one idiopathic high pressure hydrocephalus.

## Abbreviations

BBR: B cell beads rosettes
CBA: Cell-based assay
CSF: Cerebrospinal fluid
HI: Healthy individuals
MOG: Myelin oligodendrocyte glycoprotein
MS: Multiple sclerosis
RR: Relapsing-remitting
SP: Secondary progressive.
