# Supplementary material for: Decreased Frequency of Circulating Myelin Oligodendrocyte Glycoprotein B Lymphocytes in Patients with Relapsing-Remitting Multiple Sclerosis
Source: J Immunol Res. 2015 May 18;2015:673503. doi: 10.1155/2015/673503 (PMC4452172; doi:10.1155/2015/673503)
Supplement: Supplementary file 1 — In this supplementary material, complementary figures are added to provide further information and to highlight results obtained in the study. Supplementary material corresponds to 1) Reactivity of the 8-18 C5 antibody for MOG; 2) Phenotype of B cells and MOG BBR subsets in MS and HI; 3) Anti-MOG antibody detection and; 4) Anti-EBNA1 antibody detection. Each enumerated figure content is detailed in legend and refered in the main text as supplementary figure. [file 673503.f1.pdf]

Supplementary Figure 1: Reactivity of the 8-18 C5 antibody and MOG

a) - Beads were incubated with anti-MOG antibody 8-18C5 for 30 minutes and a fluorescent secondary antibody was added to reveal the MOG coated beads. Beads coated with MOG are recognized by the 8-18C5 antibody.

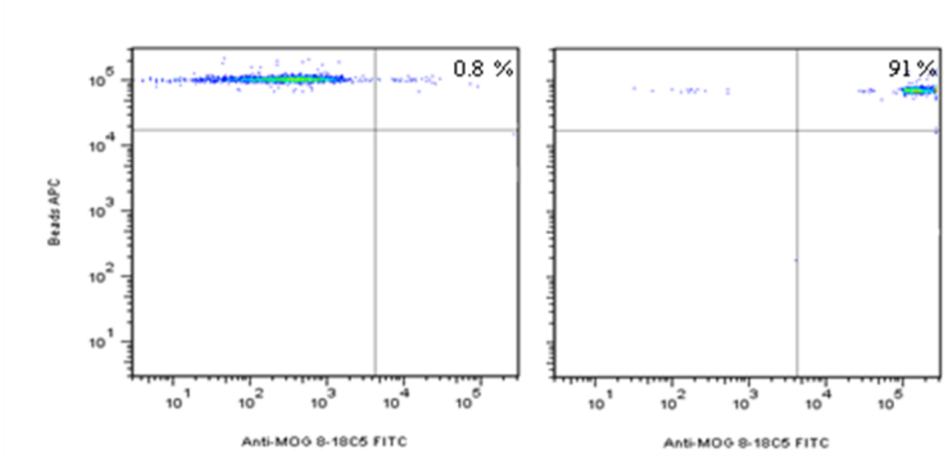

Supplementary figure 1a: Fluorescent beads were coated or not with MOG, and tested for 8-18C5 anti-MOG antibody binding. Beads without protein were not recognized by the antibody (0.8%). 91% of MOG coated beads are revealed by the 8-18C5 antibody

b) - Splenocytes from IgH-MOG transgenic mice were used to assess the potential of B cells to recognize specifically MOG coated beads. B cells which recognized MOG-Beads then were tested for the inhibition of this recognition using soluble MOG. Transgenic BCR recognized specific MOG and soluble MOG can inhibit recognition.

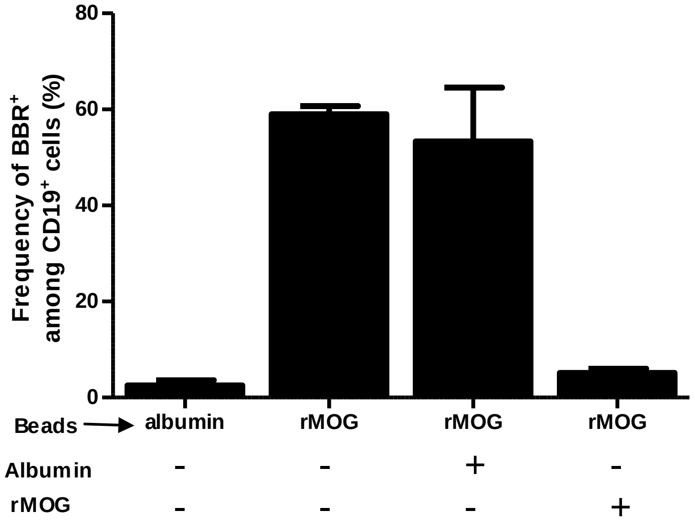

Supplementary Figure 1b: Splenocytes were incubated with MOG-beads or albumin beads. To test this recognition, 20µg of soluble MOG or soluble albumin was added before incubation with MOG-beads. Only pre-incubation with soluble MOG blocks the interaction between B cells and MOG-beads.

Supplementary Figure 2: Phenotype of B cells and MOG BBR subsets in MS and HI.

(a)

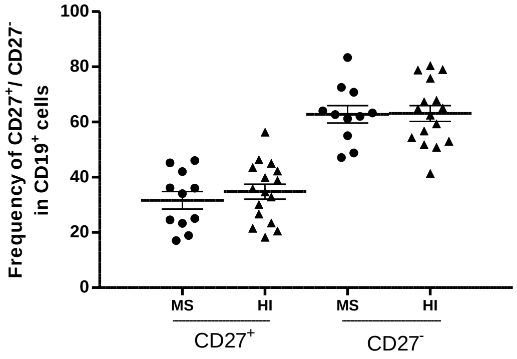

Supplementary figure 2a: B cells stained by CD19 and CD27 were incubated with MOG coated beads. Memory (CD27+) and naïve B cells (CD27-) were assessed in RRMS (n=11) and HI (n=16). No significant difference was observed between RRMS and HI for memory or naïve subsets distribution. (*Mann whitney test*, p>0.05).

(b)

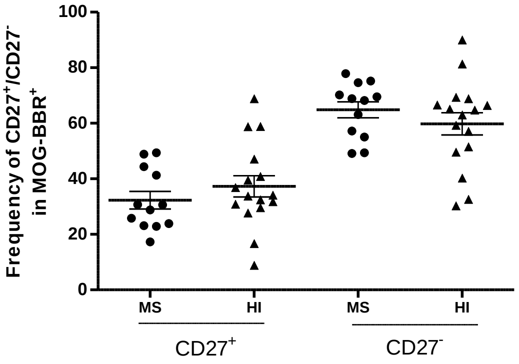

(c)

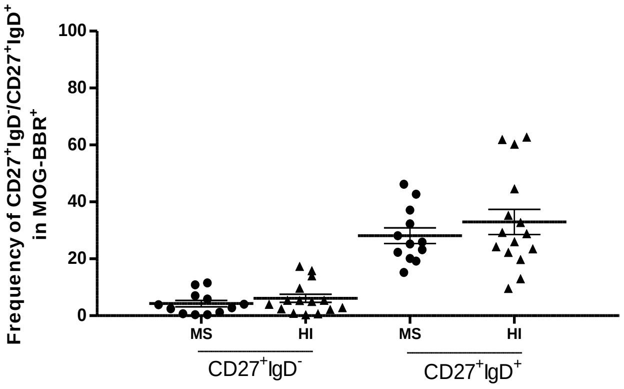

(d)

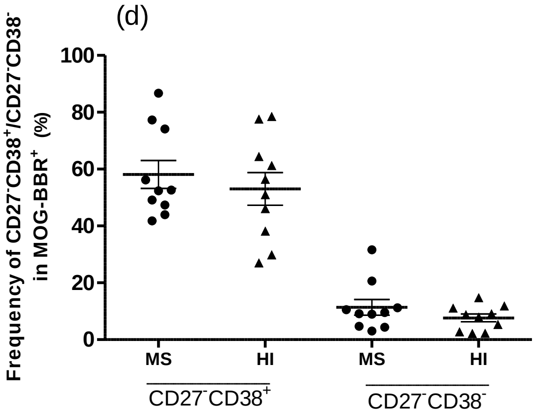

Supplementary figure 2b, 2c, 2d: B cells stained by CD19, CD27, IgD and CD38 antibodies were incubated with MOG coated beads. The frequency of memory ($CD27^+$) and naïve ($CD27^-$) MOG-BBR subset in MS (n=12) and HI (n=16) is represented. No statistical differences are observed between MS and HI in memory and naïve sub-populations (*Mann whitney test*) (**b**). The frequency of switched ($CD27^+IgD^-$) and unswitched ($CD27^+IgD^+$) memory MOG-BBR subsets was assessed in MS patients (n=12) and HI (n=15). No statistically significant difference was observed either (*Mann whitney test*) (**c**). In naïve compartment, the Frequency of $CD27^-CD38^+$ (activated-naïve B cells) and $CD27^-CD38^-$ (Mature naïve B cells) among MOG-BBR in MS patients (n=10) and HI (n=10) was represented. No difference was observed between MS and HI for each subset (*Mann whitney test*) (**d**).

(e)

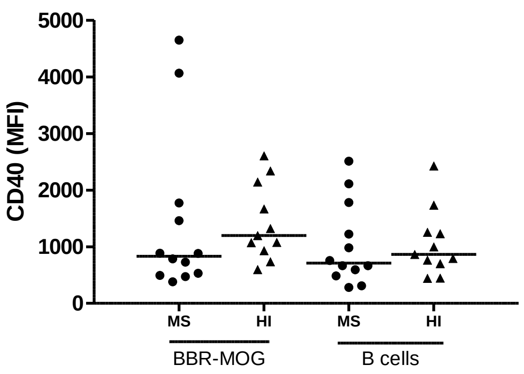

*Supplementary figure 2e***:** B cells labeled with CD40 antibody were incubated with MOG coated beads to assess the MFI of this activation marker on B cells. The fluorescent intensity of CD40 labeling in MOG-BBR subset and B cells is represented. No statistical difference was observed between MS (n=12) and HI (n=11) (*Kruskall-wallis test*).

Supplementary Figure 3: Anti-MOG antibody detection

a)- Analyses were performed as described in (Di Pauli et al. 2011).

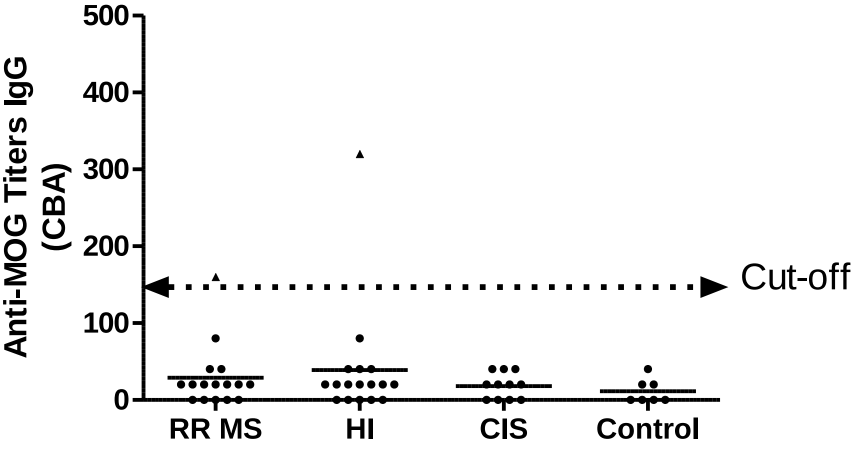

Supplementary figure 3a: Plasma samples from MS patients (n=16), HI (n=17), CIS (n=11) and controls (n=7) were tested for the presence of MOG IgG by immunofluorescence cell-based assay with human MOG expressed in HEK293 cells (positive cut-off 1:160).

b) - Correlation between the frequency of MOG-specific B cells and anti-MOG IgG titers.

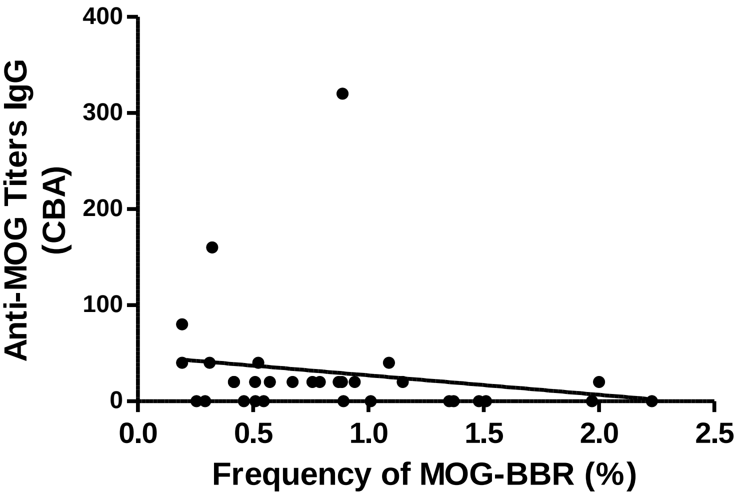

Supplementary figure 3b: The titers of MOG IgG are represented according to the frequency of MOG specific B cells for a subset of MS patients and HI. We performed the linear regression, r=0.032 for 32 values.

Supplementary Figure 4: Anti-EBNA1 antibody detection

Anti-EBNA1 IgG was detected on the plasma of MS patients (n=14) and HI (n=19) by Enzyme-Linked ImmunoSorbent Assay (ELISA).

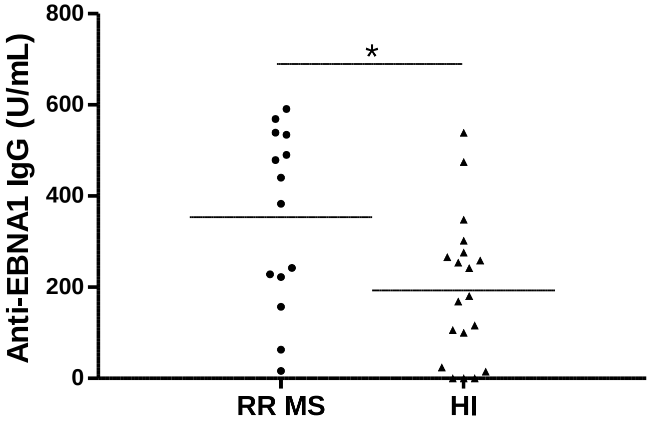

Supplementary Figure 4: The anti-EBNA1 detection was performed in two groups: MS patients with the relapsing-remitting form (n=14) and HI (n=19). There was a significant difference between RRMS and HI, (*, *Mann Whitney Test*, $p<0.05$.
